# Substantial Improvement in UK Cervical Cancer Survival with Chemoradiotherapy: Results of a Royal College of Radiologists’ Audit

**DOI:** 10.1016/j.clon.2010.06.002

**Published:** 2010-09

**Authors:** C.L. Vale, J.F. Tierney, S.E. Davidson, K.J. Drinkwater, P. Symonds

**Affiliations:** ∗ Meta-analysis Group, MRC Clinical Trials Unit, London, UK; † The Christie NHS Foundation Trust, Manchester, UK; ‡ The Royal College of Radiologists, London, UK; § Department of Cancer Studies & Molecular Medicine, University of Leicester, Leicester Royal Infirmary, Leicester, UK

**Keywords:** Audit, cervical cancer, chemoradiotherapy

## Abstract

**Aims:**

To compare survival and late complications between patients treated with chemoradiotherapy and radiotherapy for locally advanced cervix cancer.

**Materials and methods:**

A Royal College of Radiologists’ audit of patients treated with radiotherapy in UK cancer centres in 2001–2002. Survival, recurrence and late complications were assessed for patients grouped according to radical treatment received (radiotherapy, chemoradiotherapy, postoperative radiotherapy or chemoradiotherapy) and non-radical treatment. Late complication rates were assessed using the Franco-Italian glossary.

**Results:**

Data were analysed for 1243 patients from 42 UK centres. Overall 5-year survival was 56% (any radical treatment); 44% (radical radiotherapy); 55% (chemoradiotherapy) and 71% (surgery with postoperative radiotherapy). Overall survival at 5 years was 59% (stage IB), 44% (stage IIB) and 24% (stage IIIB) for women treated with radiotherapy, and 65% (stage IB), 61% (stage IIB) and 44% (stage IIIB) for those receiving chemoradiotherapy. Cox regression showed that survival was significantly better for patients receiving chemoradiotherapy (hazard ratio = 0.77, 95% confidence interval 0.60–0.98; *P* = 0.037) compared with those receiving radiotherapy taking age, stage, pelvic node involvement and treatment delay into account. The grade 3/4 late complication rate was 8% (radiotherapy) and 10% (chemoradiotherapy). Although complications continued to develop up to 7 years after treatment for those receiving chemoradiotherapy, there was no apparent increase in overall late complications compared with radiotherapy alone when other factors were taken into account (hazard ratio = 0.94, 95% confidence interval 0.71–1.245; *P* = 0.667).

**Discussion:**

The addition of chemotherapy to radiotherapy seems to have improved survival compared with radiotherapy alone for women treated in 2001–2002, without an apparent rise in late treatment complications.

## Introduction

Since 1987 the incidence of cervical cancer in the UK has fallen from around 16 to about 8 per 100 000 [1], mainly due to the successful screening campaign [2]. However, since 2000, incidence has largely plateaued. Cancer Registry data [1] showed around a third of women have locally advanced disease (tumours greater than stage I) at diagnosis. Such patients are more likely to be elderly or socio-economically deprived [3].

In the UK, patients with International Federation of Gynecology and Obstetrics (FIGO) stage IIB to IVA tumours are treated by radiotherapy. However, during the last 10 years, selected patients have also received concomitant chemotherapy. This change in practice was largely due to an unprecedented recommendation made by the US National Cancer Institute [4], as a result of five trials [5–9] showing a benefit of chemoradiotherapy in women with cervical cancer. A recent systematic review based on individual patient data from all relevant trials reported an absolute survival benefit of 6% at 5 years with chemoradiotherapy compared with the same radiotherapy alone [10]. Anecdotally this has been the experience of many UK clinical oncologists, albeit with some concern regarding an apparent increase in serious late effects [11–13].

To assess the effect of chemotherapy given with radiotherapy on survival and late effects in practice, the Faculty of Oncology of The Royal College of Radiologists carried out a national audit of outcomes of patients treated in 2001–2002 using radiotherapy or chemoradiotherapy.

## Materials and Methods

Clinical oncology audit leads or members of the Gynaecology Site Oriented e-Network of the Royal College of Radiologists from 56 National Health Service radiotherapy units and one private centre in the UK were requested to complete Snap-9 Professional web-based forms regarding all appropriate patients, either diagnosed or treated between 1 January 2001 and 31 December 2002.

### Data Available

Data on patient and tumour characteristics, treatment and outcomes were collected from 9 January until 24 December 2008. This included late complications, based on the Franco-Italian glossary [14], which had been used in a previous and similar national audit [15].

Patients were grouped according to treatment received: radical radiotherapy (at least 40 Gy external beam radiotherapy plus brachytherapy or greater than 50 Gy external beam radiotherapy with no brachytherapy); radical chemoradiotherapy (radical radiotherapy plus any concurrent chemotherapy regimen); surgery plus postoperative radiotherapy or chemoradiotherapy (any radiotherapy or chemoradiotherapy given after any surgery provided the time from diagnosis to the start of external beam radiotherapy was 6 months or less), or non-radical radiotherapy (any radiotherapy or chemoradiotherapy that did not meet the definition of radical as outlined above). Patients with missing treatment or survival data; FIGO stage IVB disease; treated outside the audit time criteria; or receiving radiotherapy only on relapse (defined as greater than 6 months’ delay between diagnosis and the start of external beam radiotherapy) were excluded from the analyses.

### Definition of Outcomes

Overall survival was defined as the time from diagnosis (at initial biopsy) until death by any cause. Disease-specific survival was defined as the time from diagnosis until death from cervical cancer. Patients alive or with unknown date of death were censored on the date of last follow-up. Rates of local recurrence (confined to the pelvis) and distant metastases (any site) were also supplied for each patient. Crude overall (any grade) late complication rates were calculated from individual types and grades of late complication. For patients who experienced more than one type, the most severe grade was used. Patients with no complications or unknown date of complications were censored on the date of last follow-up. If the date of diagnosis was unknown, the start date of external beam radiotherapy was used to calculate survival time and time to late complications.

### Analysis

Kaplan–Meier survival estimates and associated standard errors were calculated at 3 and 5 years for each treatment group and also by stage for those receiving radical radiotherapy or chemoradiotherapy. Kaplan–Meier estimates were also calculated for the time to late bladder and late bowel toxicity. No formal survival analyses were conducted for those receiving non-radical treatment, as treatment was probably with palliative (and not curative) intent. This was consistent with a previous national audit [15].

The Cox proportional hazards model (overall survival) or logistic regression model (local recurrence and distant metastases) was used to assess whether treatment was independently prognostic for outcome when other factors were taken into account. Age, FIGO stage, pelvic node involvement, size of centre, time from diagnosis until first radiotherapy treatment, whether or not the patient received brachytherapy and brachytherapy dose rate were included in univariate analyses. Those variables that were found to be significantly prognostic for outcome (*P* < 0.05) were included in multivariate analyses together with treatment group. If there were missing data for any of these variables, patients were necessarily excluded from both the univariate and multivariate analyses, further limiting the power of these analyses.

The Cox proportional hazards model was also used to assess whether treatment was independently prognostic for late complications taking into account the same factors assessed for overall survival and additional treatment-related factors that may influence late complications (total dose, fraction size and number of fractions of external beam radiotherapy and duration of all radiotherapy). Variables found to be significantly prognostic in univariate analyses (*P* < 0.05) were included in multivariate analysis together with treatment group. Again, patients were excluded from the univariate and multivariate analyses where data on these variables were not available.

## Results

### Data Available

Data were supplied on 1412 patients from 42 centres (74%) across the UK; 33 in England, three in Scotland, two in Wales and one in Northern Ireland. Three centres supplied incorrect centre codes and could not be identified. The median number of patients reported per centre was 18 (interquartile range 13–30). In total, 169 patients (12%) were excluded from the analyses because of missing data (79); extrapelvic disease (47); treatment outside the audit timeframe (29); radiotherapy given on relapse (12); or because they were duplicates (2). Data were therefore available for 1243 patients. As 168 received non-radical treatment, survival analyses are based on 1075 patients, with a median follow-up for living patients of 5.2 years.

### Patient Characteristics

Table 1 shows the distribution of age, stage and nodal involvement of patients according to treatment group. The median age at the time of diagnosis ranged from 44 to 63 years. Notably, the median age of women in the radiotherapy group (63 years) was somewhat older than in both the chemoradiotherapy (48 years) and postoperative radiotherapy groups (44 years). Overall, most women had either stage II or III disease, but in the postoperative radiotherapy group, most women (68%) had stage I disease. Furthermore, in the chemoradiotherapy group, around two-thirds of patients with stage I disease had stage Ib2, compared with less than one-third of the stage I patients in the other treatment groups. Most women in each treatment group had no pelvic lymph node involvement, but almost half of the women in the postoperative radiotherapy group (45%) had involved nodes.

### Treatment Characteristics

Table 2 shows that women mainly received three- or four-field external beam radiotherapy (40–50 Gy) and for the radical radiotherapy and chemoradiotherapy groups this was generally coupled with medium or high dose rate brachytherapy. Many women in the postoperative radiotherapy (57%) and non-radical radiotherapy (76%) groups received no brachytherapy. The median time from diagnosis to the start of external beam radiotherapy ranged from 38 days (radical chemoradiotherapy) to 71 days (postoperative radiotherapy), reflecting the obvious delay due to upfront surgery in these women. For women receiving chemoradiotherapy, the vast majority received weekly cisplatin at 40 mg/m^2^/cycle. The median number of cycles received per patient was five (range one to seven).

### Overall and Disease-specific Survival

**Fig. 1:**
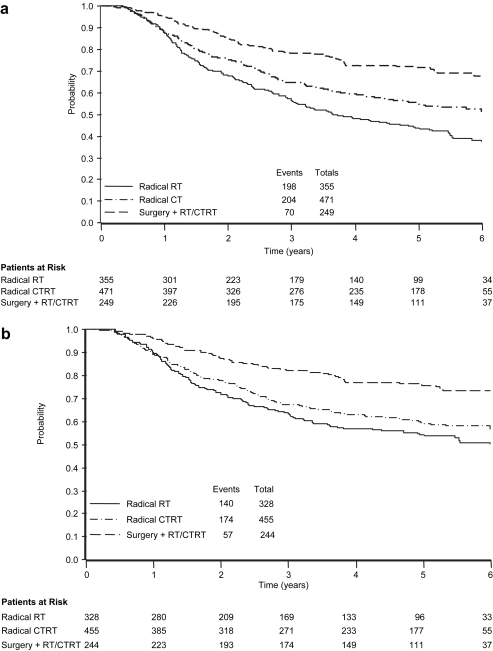
Kaplan–Meier curves for (a) overall survival and (b) cancer-specific survival.

Overall 5-year survival was 56% (median 6.2 years; standard error = 0.31 years). Further analyses were conducted within patients grouped by treatment received. However, as this was not a randomised comparison, the effect of differential patient selection (and hence prognosis) is unclear. At 5 years, overall survival was 44% (standard error = 2.9%) for radical radiotherapy; 55% (standard error = 2.6%) for radical chemoradiotherapy and 71% (standard error = 3.3%) for postoperative radiotherapy (Fig. 1a). Disease-specific survival was 54% (standard error = 3.2%) for radical radiotherapy; 59% (standard error = 2.6%) for radical chemoradiotherapy and 76% (standard error = 3.3%) for postoperative radiotherapy (Fig. 1b).

**Fig. 2:**
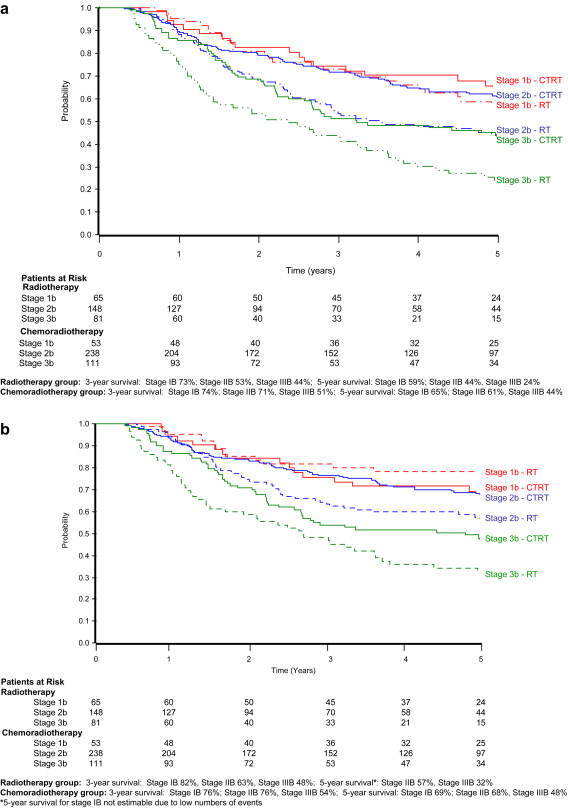
Kaplan–Meier curves for (a) overall survival and (b) disease-specific survival by tumour stage (radical radiotherapy and chemoradiotherapy groups only).

For women receiving radical radiotherapy and chemoradiotherapy, overall and disease-specific survival are also presented by stage (Fig. 2). At 3 years, survival was 73% (standard error = 6.3%), 53% (standard error = 4.3%) and 44% (standard error = 5.6%), respectively, for women with stage IB, IIB and IIIB disease receiving radical radiotherapy and 74% (standard error = 6.5%), 71% (standard error = 3.1%) and 51% (standard error = 4.9%) for those receiving radical chemoradiotherapy. At 5 years, survival by stage was 59% (standard error = 7.8%), 44% (standard error = 4.5%) and 24% (standard error = 5.0%), respectively, for those receiving radical radiotherapy and 65% (standard error = 7.8%), 61% (standard error = 3.6%) and 44% (standard error = 5.1%) for those receiving radical chemoradiotherapy (Fig. 2a). It should be noted that, in general, the results at 5 years are less reliable because of relatively low numbers of patients at risk. A similar pattern was observed for disease-specific survival (Fig. 2b).

The Cox regression for overall survival included 831 patients (348 deaths) who received radical treatment and for whom complete information on all variables included in the model was available. Table 3 shows that overall survival decreased with increasing age, increasing tumour stage and presence of involved pelvic lymph nodes. Even when these prognostic factors were taken into account, overall survival was significantly better for those receiving surgery plus postoperative radiotherapy (hazard ratio = 0.44, 95% confidence interval 0.31–0.63; *P* < 0.001) and for patients receiving radical chemoradiotherapy (hazard ratio = 0.77, 95% confidence interval 0.60–0.98; *P* = 0.037) compared with those receiving radical radiotherapy alone.

### Local Recurrence Rates

Local recurrence (confined to the pelvis) was recorded for all patients. Table 4 shows that most of the women in each treatment group had no local recurrence, with crude rates of local recurrence in the region of 20% across all treatment groups. Logistic regression was based on 891 patients (162 recurrences) who received radical treatment and for whom complete information was available. Table 5 shows that the risk of local recurrence was greater with increasing stage and involved pelvic nodes increased, but treatment received had no independent effect.

### Distant Metastases Rates

Distant metastases status was available for all except 31 patients across the four treatment groups. Again, the vast majority of women did not experience any metastases, with the crude rate of metastases across all treatment groups in the region of 20% (Table 4). The major sites of metastases (where specified) included the lymph nodes, lung, liver and bone. As there were more patients with unknown distant recurrence status, logistic regression was based on 780 patients (168 metastases) who received radical treatment and for whom complete information was available. Table 5 shows that involved pelvic lymph nodes, smaller centre size (lower numbers of patients treated per year) and high dose rate brachytherapy (compared with no brachytherapy) increased the risk of metastases. However, there was no apparent independent effect of treatment received.

### Late Toxicity

**Fig. 3:**
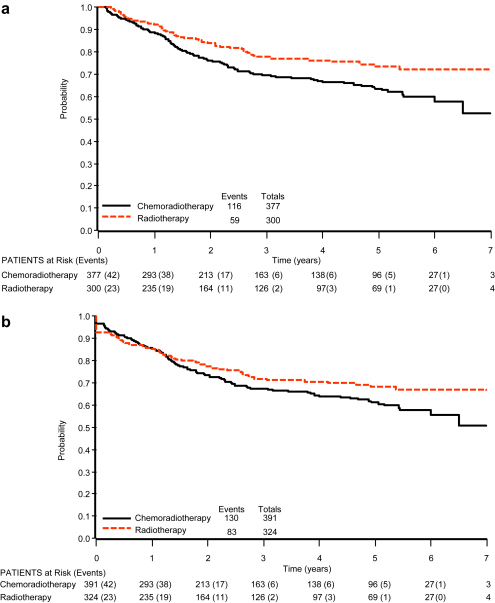
Kaplan–Meier curves for (a) late bowel toxicity and (b) overall late bladder toxicity.

The crude late complication rates were broadly similar across all treatment groups (Table 4). Ten deaths were attributed to late complications overall. However, none was recorded in the radical radiotherapy group. The major sites of serious (i.e. grade 3–4) late toxicity were the vagina, bowel and bladder (Table 4). For the chemoradiotherapy and radiotherapy groups, the most common site of serious late complications was the vagina (5% and 4%, respectively) compared with only 1% of patients in the postoperative radiotherapy group. Three years after treatment, almost all of the bowel and bladder toxicity had occurred in the radiotherapy group, in contrast with the chemoradiotherapy group, where only around half of the bowel toxicity and around 60% of bladder toxicity had occurred (Fig. 3a and b).

The Cox regression of overall late complications was based on 711 patients (369 events) who received radical treatment and for whom complete information was available. (Table 6) shows that late toxicity increased with age and decreased with increasing centre size. Stage, pelvic nodal involvement and the other treatment-related factors assessed did not independently affect late toxicity.

## Discussion

This Royal College of Radiologists’ audit shows that for patients with cervical cancer treated with curative intent in 2001–2002, the 5-year survival was 56%. Predictably, the multivariate analysis showed that stage, age and presence of pelvic lymph node metastases were the variables that affected survival. The use of chemoradiotherapy also seemed to be a significant factor in improved survival compared with radical radiotherapy, with a hazard ratio of 0.77 (*P* = 0.037), suggesting that better survival in the chemoradiotherapy group was not wholly due to patient selection. This is also in line with results of a recent systematic review of individual patient data [10] comparing chemoradiotherapy against radiotherapy (hazard ratio = 0.81). Furthermore, although chemoradiotherapy seemed to improve survival for patients with stage IIB and IIIB disease compared with those receiving radiotherapy, there seemed to be little effect in women with stage IB disease. However, this may be due to the higher proportion of stage IB2 tumours in the chemoradiotherapy group. The multivariate analysis also showed a significant improvement in survival for those patients receiving postoperative radiotherapy compared with those receiving radical radiotherapy (hazard ratio = 0.44, *P* < 0.001) with 71% survival at 5 years. This large improvement may be explained by selection of the best prognosis patients for surgery.

The only independent prognostic factors observed for local recurrence were stage and involved pelvic nodes, and for distant metastases, size of centre, involved pelvic nodes and high dose rate brachytherapy. The effect of brachytherapy dose rates might simply be reflecting that only the best prognosis or lowest risk patients do not receive brachytherapy, but the suggestion that local recurrence and distant metastases are unaffected by the treatment received is surprising, given the improvement in both local and distant recurrence with chemoradiotherapy noted in a recent systematic review [10] and the effect on survival seen in this audit. However, local and distant recurrence rates in the audit were around 20% compared with rates in the region of 40–50% in the systematic review [10] and with FIGO data [16]. Although it is not clear if this is due to underreporting or for other reasons, it does mean that there are about 50% fewer events available for the multivariate analyses of recurrence than for survival. Furthermore, as the dates of recurrence were not collected, we could only examine the effect of different factors on the rates of recurrence and not the time to recurrence. Both of these differences may have limited the power and sensitivity of our multivariate analyses to reliably detect anything other than the strongest predictors of recurrence and, therefore, these results should be viewed with some caution.

The increase in survival with chemoradiotherapy seems to be achieved with no apparent increase in overall late toxicity, although there may again be an issue with power. There were six deaths attributed to late complications in the chemoradiotherapy group, including four due to late bowel complications, and none in the radiotherapy group. The bowel may be infiltrated and fixed by the tumour predisposing it to perforation during or after radiotherapy. This may be exacerbated in the chemoradiotherapy patients, where there is a greater response to treatment. Alternatively, there may be an additive effect of the chemotherapy on the vasculature of the bowel. The most common site of serious late complications was the vagina. If the vagina is heavily involved with tumour, as is often the case with advanced disease, healing will be with fibrosis leading to stenosis. The incidence of serious late complications in the postoperative group was less than expected, possibly reflecting the fact that only 44% of this group received brachytherapy. Although almost half the patients included in the audit had some degree of toxicity, data rely on patient records, where only the recording of serious morbidity has been found to be reliable [17]. It is therefore possible that grade 1–2 late complications are under-represented [18]. Interestingly, these results seem to suggest reduced levels of late complications (and metastases) in the larger radiotherapy centres. Our exploratory analyses (data not shown) seem to support the current guidance of the Royal College of Radiologists that centres should aim to treat at least 50 patients with gynaecological malignancies per year using brachytherapy.

The time frame of treatment and diagnosis for this audit should provide sufficient follow-up to allow for the development of any late complications, and there does seem to be a difference in the time to development of late complications between the treatment groups. In the radiotherapy group, the vast majority of late bowel and bladder complications occurred within the first 3 years after treatment. However, in the chemoradiotherapy group, complications seem to still be developing up to 7 years after treatment. Few of the randomised trials have reported late treatment complications after chemoradiotherapy and indeed a recent systematic review of individual patient data from all randomised trials [10] could not formally assess the effect of treatment on late toxicity due to the lack of data available.

Using UK Cancer Registry data, we estimate 1800–2000 eligible cases of cervical cancer during the audit timeframe. We obtained data for 1412 women, consistent with three-quarters of all centres having participated. However, around 12% of these were excluded from all of the analyses, with up to an additional 364 patients being excluded from the multivariate analyses, due to missing outcome or baseline data. Clearly, we cannot be certain how exclusion of these patients or those from centres not participating in the audit might affect these results, but certainly their omission may introduce bias.

Probably the best yardstick to judge these results is the national audit of patients treated in 1993 by radiotherapy [15], where the 5-year survival for all patients was 47%. Thus, in the 8 years between these two audits, there has been a potential 9% improvement in 5-year survival. Overall survival during this period also seems to have improved for patients with locally advanced disease, particularly those receiving chemoradiotherapy. In 2001–2002, radiotherapy or chemoradiotherapy would have been the preferred treatment for most women, except those eligible for radical surgery, with extrapelvic spread (stage IVB), the very frail or elderly. However, practice was changing at that time. Although some centres had begun to use chemoradiotherapy, many were still using radiotherapy alone, largely because it could be given in the outpatient setting. Survival for stage IIIB was 23% in 1993 compared with 44% for those receiving chemoradiotherapy in 2001–2002. Similarly, 5-year survival for stage IIB disease was 47% in 1993 compared with 63% after chemoradiotherapy in 2001–2002. However, comparisons of survival between 1993 and 2001–2002 should be interpreted with caution due to somewhat limited data at 5 years. Also, since 1993, more sophisticated radiology, including computed tomography and magnetic resonance imaging scanning, has resulted in improved diagnosis of stage IVB disease, resulting in more of the poorest prognosis patients being excluded from the current audit. The patient groups within the two audit series also differ. For example, women treated with chemoradiotherapy or postoperative radiotherapy in 2001–2002 were younger, and those receiving radiotherapy were older at the time of diagnosis than those in the 1993 series. Furthermore, in 1993, 46.1% of women had stage IA–IIA disease, compared with only 19% (chemoradiotherapy) and 27% (radiotherapy) in 2001–2002. This is probably because patients with lower stage disease are now more commonly treated with surgery.

The treatment of locally advanced cervical cancer remains a challenge. Current experimental approaches include the addition of hypoxic cell sensitisers, such as tirapazamine, or inhibitors of angiogenesis, such as bevacizumab, to chemoradiotherapy. However, the results of this audit show that when compared with radiotherapy alone, concomitant chemoradiotherapy (mainly weekly cisplatin) seems to have improved survival of women with locally advanced disease. Wider experience and increased use of chemoradiotherapy in the UK since 2002 may further improve survival for women with locally advanced cervical cancer.

### Role of the Funding Source

The Royal College of Radiologists sponsored this project. Karl Drinkwater is the Audit Officer for the Royal College of Radiologists and acted as the central data collection point for the audit. Data for the audit were supplied by unpaid Fellows of the Royal College of Radiologists from 42 centres across the UK. Paul Symonds and Susan Davidson are Fellows of the Royal College of Radiologists. Claire Vale and Jayne Tierney are employed and funded by the UK Medical Research Council. The sponsor had no other involvement in the study design; collection, analysis and interpretation of data or in the decision to submit this paper for publication.

## Figures and Tables

**Table 1:** Patient characteristics

	Radical radiotherapy (355 patients)	Radical chemoradiation (471 patients)	Radical surgery + postoperative radiotherapy (249 patients)	Non-radical treatment (168 patients)
Age (years)				
≤35	13 (5%)	66 (15%)	52 (22%)	7 (5%)
36–50	49 (19%)	178 (41%)	99 (42%)	32 (24%)
51–65	85 (33%)	136 (31%)	64 (27%)	33 (25%)
>65	113 (43%)	56 (13%)	18 (8%)	60 (46%)
Unknown	95	35	16	36
Median age	62.50	48.00	44.00	63.00
Range	21–88	18–81	21–82	24–89
Stage				
IA	0	0	2 (1%)	2 (1%)
IB∗	14 (4%)	4 (1%)	44 (18%)	12 (8%)
IB1	30 (9%)	15 (3%)	75 (30%)	8 (5%)
IB2	21 (6%)	34 (7%)	47 (19%)	5 (3%)
IIA	26 (7%)	34 (7%)	22 (9%)	6 (4%)
IIB	148 (43%)	238 (51%)	37 (15%)	36 (23%)
IIIA	13 (4%)	14 (3%)	2 (1%)	11 (7%)
IIIB	81 (23%)	111 (24%)	12 (5%)	43 (28%)
IVA	14 (4%)	19 (4%)	2 (1%)	31 (20%)
Unknown	8	2	6	14
Pelvic node involvement				
Not involved	224 (73%)	284 (72%)	129 (55%)	85 (64%)
Involved	83 (27%)	109 (28%)	106 (45%)	48 (36%)
Unknown	48	78	14	35

∗Unspecified substage.

**Table 2:** Treatment characteristics

	Radical radiotherapy (355 patients)	Radical chemoradiotherapy (471 patients)	Surgery + postoperative radiotherapy (249 patients)	Non-radical treatment (168 patients)
Surgery				
No surgery	257 (98%)	419 (96%)	0	112 (94%)
Hysterectomy	0	1 (<1%)	204 (82%)	3 (3%)
Laparotomy	0	4 (<1%)	23 (9%)	0
Trachelectomy + lymphadenectomy	0	0	1 (<1%)	0
Pelvic lymphadenectomy	0	0	1 (<1%)	0
Salvage surgery	4 (2%)	13 (3%)	1 (<1%)	3 (3%)
Unspecified type	1 (<1%)	0	19 (8%)	1 (<1%)
Unknown	93	34	0	49
External radiotherapy type				
No radiotherapy	0	0	0	11 (7%)
Three or four field	276 (78%)	393 (83%)	197 (79%)	87 (52%)
Parallel opposed	21 (6%)	41 (9%)	8 (3%)	47 (28%)
Three or four field and parallel opposed	0	3 (1%)	0	0
Conformal radiotherapy	50 (14%)	15 (3%)	33 (14%)	13 (8%)
Parallel opposed and conformal	0	1 (<1%)	0	2 (1%)
Unspecified type	8 (2%)	18 (4%)	11 (4%)	8 (5%)
External radiotherapy dose				
No external radiotherapy	0	0	0	11 (7%)
<40 Gy	0	0	13 (5%)	70 (42%)
40–50 Gy	317 (89%)	435 (92%)	230 (92%)	87 (52%)
>50 Gy	38 (11%)	36 (8%)	6 (2%)	0
Brachytherapy				
Yes	319 (90%)	448 (95%)	109 (44%)	41 (24%)
No	36 (10%)	23 (5%)	140 (56%)	127 (76%)
Brachytherapy dose rate				
High	132 (38%)	151 (32%)	38 (15%)	7 (4%)
Medium	148 (42%)	208 (44%)	55 (22%)	25 (15%)
Low	39 (11%)	89 (19%)	14 (6%)	8 (5%)
No brachytherapy	36 (10%)	23 (5%)	140 (57%)	127 (76%)
Unknown	1	0	2	1
Chemoradiotherapy regimen				
No chemoradiotherapy	398 (100%)	0	154 (62%)	125 (74%)
Cisplatin (single agent)	0	447 (95%)	85 (34%)	36 (21%)
Cisplatin/5-fluorouracil	0	3 (1%)	8 (3%)	0
Cisplatin/methotrexate/bleomycin	0	5 (1%)	0	3 (2%)
Carboplatin	0	11 (2%)	1 (<1%)	2 (1%)
Other	0	4 (1%)	1 (<1%)	2 (1%)
Time from diagnosis to start of external beam radiotherapy				
Median (days)	48	38	71	39
Interquartile range (days)	35–66	27–52	47–97	25–58

**Table 3:** Multivariate Cox regression analysis of overall survival

Covariate	B	Degrees of freedom	*P*-value	Hazard ratio (95% confidence interval)
Overall survival (831 patients)				
Age	0.009	1	0.026	1.01 (1.00–1.02)
Stage				
Stage I	–	–	–	–
Stage II	0.235	1	0.141	1.26 (0.93–1.73)
Stage III–IVA	0.760	1	<0.001	2.14 (1.54–2.98)
Pelvic node involvement				
Not involved	–	–	–	–
Involved	0.625	1	<0.001	1.87 (1.49–2.34)
Treatment category				
Radiotherapy	–	–	–	–
Chemoradiotherapy	−0.267	1	0.037	0.77 (0.60–0.98)
Surgery + postoperative radiotherapy	−0.817	1	<0.001	0.44 (0.31–0.63)

**Table 4:** Survival, recurrence and late complication rates (with site of grade 3/4 complication)

	Radical radiotherapy (355 patients)	Radical chemoradiation (471 patients)	Surgery + postoperative radiotherapy (249 patients)	Non-radical treatment (168 patients)
Follow-up				
Median follow-up (range in years)	5.1 (0.10–7.9)	5.2 (0.15–7.9)	5.2 (0.20–7.4)	4.8 (0.10–7.5)

Survival				
Alive	157 (44%)	267 (57%)	79 (72%)	–
Dead	198 (56%)	204 (43%)	70 (28%)	–
Dead (due to cancer)	140 (39%)	174 (37%)	57 (23%)	–
Median overall survival	3.7 years	6.4 years	>7.4 years	
Median cancer-specific survival	6.2 years	>7.9 years	>7.4 years	

Local (pelvic) recurrence				
No recurrence	286 (81%)	366 (78%)	218 (88%)	122 (73%)
Recurrence	69 (19%)	105 (22%)	31 (12%)	46 (27%)
Unknown	0	0	0	0

Distant metastases				
No metastases	275 (81%)	339 (73%)	205 (85%)	135 (83%)
Metastases	66 (19%)	128 (27%)	36 (15%)	28 (17%)
Unknown	14	4	8	5

Overall late complications (crude rates)				
Grade 1–2	142 (43%)	200 (47%)	88 (41%)	35 (24%)
Grade 3–4	27 (8%)	44 (10%)	10 (5%)	13 (9%)
No toxicity	162 (49%)	178 (42%)	115 (54%)	97 (67%)
Unknown	24	49	36	23
Deaths	0	6	1	3

Main sites of complications				
Vagina				
Grade 3	11 (4%)	17 (5%)	2 (1%)	1 (1%)
Grade 4	0	0	0	0
Rectum				
Grade 3	6 (2%)	10 (3%)	2 (1%)	1 (1%)
Grade 4	0	0	0	0
Colon				
Grade 3	3 (<1%)	7 (1.5%)	1 (<1%)	1 (1%)
Grade 4	0	2 (<1%)	1 (<1%)	1 (1%)
Small bowel				
Grade 3	1 (<1%)	3 (1%)	3 (1%)	0
Grade 4	0	2 (<1%)	0	0
Bladder				
Grade 3	5 (2%)	9 (2%)	1 (<1%)	5 (4%)
Grade 4	0	0	0	0

**Table 5:** Multivariate logistic regression analysis of local recurrence and distant metastases

Covariate	B	Degrees of freedom	*P*-value	Hazard ratio (95% confidence interval)
Local recurrence (891 patients)				
Age	0.000	1	0.783	1.00 (0.999–1.001)
Stage				
Stage I	–	–	–	–
Stage II	0.524	1	0.058	1.689 (0.983–2.902)
Stage III–IVA	1.063	1	<0.001	2.894 (1.619–5.174)
Pelvic node involvement				
Not involved	–	–	–	–
Involved	0.471	1	0.012	1.601 (1.110–2.311)
Time from diagnosis to start of external beam radiotherapy	−0.005	1	0.117	0.995 (0.988–1.001)
Treatment category				
Radiotherapy	–	–	–	–
Chemoradiotherapy	−0.010	1	0.960	0.990 (0.657–1.490)
Surgery + postoperative radiotherapy	−0.220	1	0.465	0.802 (0.444–1.448)

Distant metastases (780 patients)				
Age	0.004	1	0.568	1.004 (0.990–1.019)
Stage				
Stage I	–	–	–	–
Stage II	−0.066	1	0.805	0.936 (0.555–1.579)
Stage III–IVA	0.404	1	0.170	1.498 (0.841–2.667)
Pelvic node involvement				
Not involved	–	–	–	–
Involved	1.124	1	<0.001	3.076 (2.0374–4.561)
Time from diagnosis to start of external beam radiotherapy	−0.006	1	0.080	0.994 (0.987–1.001)
Centre size	−0.011	1	<0.001	0.989 (0.986–0.993)
Brachytherapy dose rate				
No brachytherapy	–	–	–	–
Low/medium dose rate	0.369	1	0.249	1.446 (0.772–2.708)
High dose rate	0.994	1	0.003	2.703 (1.416–5.159)
Treatment category				
Radiotherapy	–	–	–	–
Chemoradiotherapy	0.031	1	0.906	1.032 (0.618–1.722)
Surgery + postoperative radiotherapy	−0.167	1	0.632	0.846 (0. 427–1.676)

**Table 6:** Multivariate Cox regression analysis of overall late complications

Covariate	B	Degrees of freedom	*P*-value	Hazard ratio (95% confidence interval)
Overall late toxicity (711 patients)				
Age	0.010	1	0.012	1.010 (1.002–1.019)
Stage				
Stage I	–	–	–	–
Stage II	0.027	1	0.848	1.028 (0.776–1.360)
Stage III–IVA	−0.014	1	0.934	0.986 (0.703–1.383)
Pelvic node involvement				
Not involved	–	–	–	–
Involved	0.153	1	0.200	1.166 (0.922–1.474)
Centre size	−0.002	1	0.004	0.998 (0.996–0.999)
Total dose of external radiotherapy	0.018	1	0.171	1.018 (0.992–1.045)
Dose per fraction (external radiotherapy)	−0.406	1	0.258	0.666 (0.329–1.347)
Brachytherapy dose rate				
No brachytherapy	–	–	–	–
Low/medium dose rate	0.297	1	0.108	1.345 (0.937–1.932)
High dose rate	0.243	1	0.218	1.275 (0.866–1.875)
Treatment category				
Radiotherapy	–	–	–	–
Chemoradiotherapy	−0.062	1	0.667	0.940 (0.710–1.245)
Surgery + postoperative radiotherapy	−0.206	1	0.270	0.814 (0.565–1.173)
